# Low incidence of primary immunodeficiency-associated cancers in children at a tertiary care pediatric hospital in Pakistan: a blessing in disguise or wet behind the ears?

**DOI:** 10.3332/ecancer.2024.1733

**Published:** 2024-07-30

**Authors:** Rahat Ul Ain, Mahwish Faizan

**Affiliations:** Department of Pediatric Hematology/Oncology and Bone Marrow Transplant, University of Child Health Sciences, The Children’s Hospital, Lahore 54600, Pakistan

**Keywords:** incidence, immune deficiency, pediatric oncology, cancers, children, low-middle-income countries

## Abstract

Scarce data is available regarding primary immunodeficiency-associated cancers in children in low-middle-income countries. This study aimed to determine the incidence, clinical features and outcomes of primary immunodeficiencies (PIDs)-associated cancers in children presenting to Pakistan’s largest public-sector specialised pediatric oncology center. Among 5,748 children with cancers registered over 5 years, only eight patients were found to have PID-associated pediatric malignancies with an incidence of 1.4 per 1,000 cases. The median age at the time of diagnosis was 6.5 years with a male-to-female ratio of 7:1. Only four types of PIDs were found to be associated with cancer in children at our center: Ataxia Telangiectasia in 37.5% (*n* = 3), hyper-IgE syndrome and IgG deficiency in 25% (each *n* = 2) and one case (12.5%) of common variable immune deficiency. Six different types of pediatric cancers were associated with PID with a predisposition towards hematological malignancies (*n* = 7, 87.5%). Only two patients (25%) survived. The median survival of the cohort was 3.5 months. Infection-related mortality was the cause of death in four patients (66%), and the type of PID was the only statistically significant factor associated with the outcome. It is concluded that a lesser proportion of PID-associated pediatric cancers are found in our center as compared to the reported data from high-income countries. PID-associated cancers in children have an abysmal prognosis and infection-related mortality is the major cause of treatment failure. Sensitisation of oncologists to look for any underlying PID, the introduction of PID-screening programs in children and consideration of PID-associated malignancies as a high-risk group for treatment may help improve the outcomes.

## Background

Primary immunodeficiencies (PIDs) also known as inborn errors of immunity (IEIs) are a group of inherited immune system disorders associated with increased susceptibility to infections, autoimmunity, allergy and cancer [1]. More than 250 different types of PIDs/IEIs have been described so far [2]. The failure in the regulation of the immune system leads to multiple diseases due to the absence of cytokines, impaired function of the B or T lymphocytes, lack of functioning proteins, or alteration in receptors [3]. Tumour cell antigens induce immune responses that destroy tumour cells developed in the body therefore the immunological approach is being increasingly used not only for the diagnosis but also in the treatment of cancer [4]. Children with primary immunodeficiency have an estimated overall risk of 4%–25% of developing malignancy [5]; therefore, it is important to recognise this condition but minimal data is available from developing countries in this regard. This study aimed to determine the incidence, clinical features and outcomes of PID-associated cancers in children presenting to Pakistan’s largest public-sector specialised pediatric oncology center.

## Methods

An ambi-directional cohort study was conducted in the Department of Pediatric Hematology/Oncology at the University of Child Health Sciences, The Children’s Hospital Lahore, Pakistan. After the approval from the institutional ethical committee, the data were collected retrospectively from January 2017 to August 2020 and prospectively from September 2020 to December 2021, with a total period of 5 years. The study included all the children with pediatric malignancies diagnosed with underlying primary immunodeficiency, aged less than 16 years. Patients with Down syndrome, Fanconi anemia and Histiocytosis syndromes were excluded. After the completion of data collection in December 2021, the study cohort was followed up for 2 years till December 2023 for survival analysis. The data were analysed in terms of descriptive statistics with SPSS version 20.0. The survival analysis was done using the Kaplan–Meier and log-rank tests. The chi-square test was applied for the determination of *p*-values.

*Primary Immunodeficiency:* Patients with primary immunodeficiency were defined as having a clinical diagnosis of one of the genetic syndromes associated with innate immune system defects.

## Results

A total of 5,748 pediatric patients with different malignant disorders were registered in the department over the 5 years study period (From January 2017 till December 2021), while only 8 patients were found to have an underlying PID with an incidence of 1.4 cases/1,000 pediatric cancer cases. The median age of presentation of patients with PID-associated malignancies was 6.5 years (3–16 years) with the male to female ratio of 7:1.

Only four types of PIDs were found to be associated with cancer in children at our center: Ataxia Telangiectasia (AT) in 37.5% (*n* = 3), Hyper-IgE syndrome and IgG deficiency in 25% each (*n* = 2 each) and one case (12.5%) of common variable immune deficiency (CVID). Similarly, six different types of malignant disorders were found to be associated with children with PID: B-cell acute lymphoblastic leukemia (ALL) (25%, *n* = 2), T-cell ALL (25%, *n* = 2), Philadelphia positive ALL (12.5%, *n* = 1), NK-cell ALL (12.5%, *n* = 1), Hodgkin lymphoma (HL) (12.5%, *n* = 1) and hepatocellular carcinoma (12.5%, *n* = 1) (Table 1).

**Figure 1.**
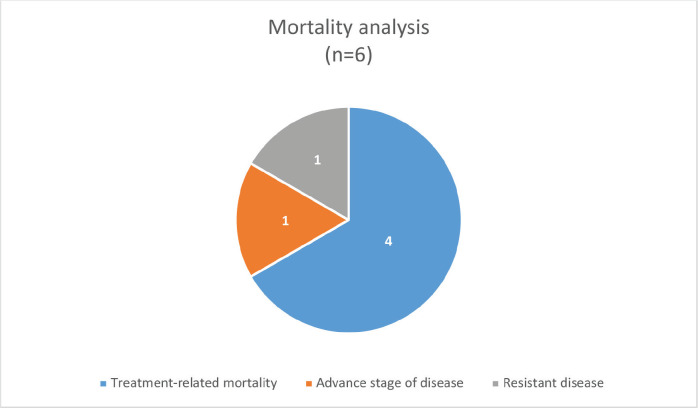
Mortality analysis of PID-associated pediatric cancers.

The median survival was 3.5 months (0.25–30 months) with a survival rate of 25% (*n* = 2, one patient completed treatment and one is under treatment) (Figure 1). The type of PID was the only significant factor associated with the outcome (*p-*value: 0.046).

## Discussion

The most common PID, CVID is associated with an increased risk of B-cell non-HL and gastric cancer but these rarely develop in childhood. Similarly, hyper-IgE syndrome also has a higher risk of developing aggressive B cell lymphomas. Chronic mucocutaneous candidiasis is associated with squamous cell cancers, and AT with the highest risk for malignancy among all PID, leukemia and lymphomas is frequently observed [6].

As compared to the published literature, mainly from the high-income countries (HICs) [3, 5, 7–10], a strikingly lesser proportion of PID-associated pediatric malignancies is found in this study. This either can be due to a lesser incidence of PID in our region or a lack of diagnosis of all PID cases. The former seems to be less likely as our country has a high rate of consanguinity and it is expected to have a higher proportion of all autosomal recessive disorders in the population, while the latter seems to be more likely the cause of this finding in our setting. There could be multiple reasons behind the lack of diagnosis of all cases of PID among the children presenting with cancer in our hospital. In this study, though a large number of pediatric oncology patients were registered during the study period, all of these patients did not go through any screening test for immune deficiency on presentation with cancer. Previous history of repeated infections was not taken into the record and definitive diagnostic tests (genetic testing) for primary immunodeficiency are not available in our setting. PIDs were rather diagnosed later during treatment in all patients except one who had a known case of AT. The diagnosis of PID was based on the recognition of specific clinical features (phenotypic abnormalities) of various immune deficiency syndromes or the development of resistant/overwhelming infections during treatment. Further diagnosis was augmented with the immunoglobulin levels. As the tests for immunoglobulin levels were done post-chemotherapy in the majority of the cases; therefore, it was made sure to have bone marrow recovery with a normal white cell count, absolute neutrophil count and absolute lymphocyte count while checking the sample for immunoglobulin levels. There is also a lack of screening programs at birth for genetic disorders in our country so despite having a large number of cases with pediatric cancers, many cases of PID would have been missed being included in the study and may lead to a smaller number of reported cases of PID-associated pediatric malignancies at our center.

A different clinical spectrum of PID-associated pediatric malignancies is also noted in this study. As compared to the previous studies [3, 5, 7–9] from HIC, the types of PIDs, types of associated cancers and the median age of presentation all are different. Non-HL has been reported to be the most common malignancy, and CVID is the most common PID with a median age of 7.1 years on presentation. Filipovich *et al* [7] found male predominance, contributed to several X-linked PIDs as well as the surprising observation of excess tumours in males with AT, an autosomal recessive disorder. In this study, male preponderance has also been observed but none of the X-linked PIDs have been found, and the patients with AT did not show any sex predilection. The possible explanation for these distinctive findings and probably the limitations to this study are that this study has been conducted among oncology patients in the pediatric age group (<16 years) only, with a small number of PID cases recognised in the study cohort resulting in a very small sample size to be analysed for. Future studies with a larger cohort and improved diagnostics for PIDs should be conducted from our region.

The outcome of PID-associated pediatric cancers in the setting of a low-middle income country (LMIC) is poor with a substantial number of patients having infection/treatment-related mortality. Primary immunodeficiency with chemotherapy-induced secondary immunodeficiency, along with the underlying canvas of suboptimal supportive care in LMIC settings adds up to these challenges. To combat these challenges in resource-limited settings it is suggested that this group of patients should be considered as a high-risk group during risk stratification with consideration of vigilant supportive care during treatment. This can be done in the form of enhanced infection prevention and control measures, and extended infection chemoprophylaxis (including not only against pneumocystis jiroveci pneumonia, but also antifungal antiviral, and antibiotic prophylaxis). Similarly, granulocyte colony-stimulating factors and intravenous immunoglobulin should be considered among the first-line therapy in the management of febrile neutropenia.

Management of children with a combination of PID and cancer is particularly challenging in a resource-limited setting where there are suboptimal healthcare facilities and the absence of screening programs for genetic disorders at birth. A substantial number of genetic disorders remain undiagnosed and present later on in life with various complications. Timely diagnosis of these disorders can not only benefit in terms of the patient’s outcomes but also provide the opportunity to screen close family members and offer appropriate management and surveillance. The oncologists working in LMIC should at least make a habit of taking the previous history of repeated infections, family history and evaluation of baseline immunoglobulin levels, in patients presenting with malignant disorders. These measures are cost-effective and feasible in resource-limited settings and can help diagnose, not all, but a substantial number of patients with cancers with PIDs.

## Conclusion

A lesser proportion of PID-associated pediatric malignancies have been found as compared to the data from HICs. Either primary immunodeficiency rarely presents with cancers in childhood in our region or it is under-diagnosed in resource-limited settings, it is yet to be investigated further. PID-associated pediatric cancers have an abysmal prognosis and infection-related mortality is the major cause of treatment failure. Sensitisation of pediatric oncologists regarding baseline assessment for PIDs in all children with cancer, the introduction of PID-screening programs in children in the country, and consideration of PID-associated pediatric malignancies as a high-risk group for treatment may help improve the outcomes.

## List of abbreviations

ALL, Acute lymphoblastic leukemia; AT, Ataxia-Telangiectasia; CVID, Common variable immune deficiency; HL, Hodgkin lymphoma; LMIC, Low/middle-income country; PID, Primary Immunodeficiency; TRM, Treatment-related mortality.

## Conflicts of interest

The authors have no potential conflicts of interest to disclose.

## Funding

The authors have not received any funding for this study.

## Author contributions

RUA: Conception, data collection, data analysis and manuscript writing.

MF: Data analysis and manuscript writing.

## Figures and Tables

**Table 1.** Characteristics of the children with PID-associated cancers.

Sr. No.	Age (years)	Gender	Malignancy	PID	Clinical manifestations	Outcome	Survival (months)	TRM	Reason for expiry
1.	6	M	T-cell ALL	AT	Ataxia and ocular telangiectasia	Expired (post-induction)	2	Yes	Invasive fungal infection
2.	10	F	Hepatocellular carcinoma	AT	Ataxia and ocular telangiectasia	Expired (post-op)	1.5	Yes	Surgical site infection
3.	16	M	Classical HL	Hyper-IgE syndrome	Invasive fungal infection during treatment not responding to therapy along with coarse facies	On treatment	28	No	Resistant / Progressive disease
4.	7	M	NK cell acute leukemia	AT	Ataxia and ocular telangiectasia	Expired (before the start of treatment)	0.25	No	Advanced disease
5.	3	M	B cell ALL	IgG deficiency	Repeated overwhelming infections not responding to treatment	On consolidation	30	-	-
6.	4	M	T-cell ALL	Hyper-IgE syndrome	Repeated skin and pulmonary infections	Expired (post-DI)	4	Yes	CMV reactivation and anal abscess
7.	4	M	Philadelphia positive ALL	IgG deficiency	Repeated overwhelming infections not responding to treatment	On treatment	25	-	-
8.	11	M	B-cell ALL	CVID	Repeated overwhelming infections not responding to treatment	Expired (during consolidation)	3	Yes	Fungal pneumonia and meningoencephalitis

PID: Primary Immunodeficiency, TRM: Treatment-related mortality, ALL: Acute lymphoblastic leukemia, AT: Ataxia-Telangiectasia, NK: Natural killer, CVID: Common Variable Immune Deficiency, DI: delayed intensification phase of treatment
